# A *Haemophilus* sp. dominates the microbiota of sputum from UK adults with non-severe community acquired pneumonia and chronic lung disease

**DOI:** 10.1038/s41598-018-38090-5

**Published:** 2019-02-20

**Authors:** Daniel G. Wootton, Michael J. Cox, Gregory B. Gloor, David Litt, Katja Hoschler, Esther German, Joanne Court, Odiri Eneje, Lynne Keogan, Laura Macfarlane, Sarah Wilks, Peter J. Diggle, Mark Woodhead, Miriam F. Moffatt, William O. C. Cookson, Stephen B. Gordon

**Affiliations:** 1 Institute of Infection and Global Health, University of Liverpool, Liverpool, UK; 2 Department of Respiratory Research, Aintree University Hospital NHS Foundation Trust, Liverpool, UK; 3 Section of Genomic Medicine, National Heart and Lung Institute, Imperial College London, London, UK; 4 Departments of Biochemistry and Applied Mathematics, University of Western Ontario, Ontario, ON Canada; 5 Respiratory and Vaccine Preventable Bacteria Reference Unit, National Infection Service, Public Health England, London, UK; 6 Virus Reference Department, National Infection Service, Public Health England, London, UK; 7 Department of Clinical Sciences, Liverpool School of Tropical Medicine, Liverpool, UK; 8 CHICAS, Lancaster University Medical School, Lancaster University, Lancaster, UK; 9 Department of Respiratory Medicine, Central Manchester University Hospitals NHS Foundation Trust, Manchester, UK; 10 Manchester Academic Health Science Centre and Faculty of Medical and Human Sciences, University of Manchester, Manchester, UK; 11 The Malawi Liverpool Wellcome Trust Clinical Research Programme, Blantyre, Malawi

## Abstract

The demographics and comorbidities of patients with community acquired pneumonia (CAP) vary enormously but stratified treatment is difficult because aetiological studies have failed to comprehensively identify the pathogens. Our aim was to describe the bacterial microbiota of CAP and relate these to clinical characteristics in order to inform future trials of treatment stratified by co-morbidity. CAP patients were prospectively recruited at two UK hospitals. We used 16S rRNA gene sequencing to identify the dominant bacteria in sputum and compositional data analysis to determine associations with patient characteristics. We analysed sputum samples from 77 patients and found a *Streptococcus* sp. and a *Haemophilus* sp. were the most relatively abundant pathogens. The *Haemophilus* sp. was more likely to be dominant in patients with pre-existing lung disease, and its relative abundance was associated with qPCR levels of *Haemophilus influenzae*. The most abundant *Streptococcus* sp. was associated with qPCR levels of *Streptococcus pneumoniae* but dominance could not be predicted from clinical characteristics. These data suggest chronic lung disease influences the microbiota of sputum in patients with CAP. This finding could inform a trial of stratifying empirical CAP antibiotics to target *Haemophilus* spp. in addition to *Streptococcus* spp. in those with chronic lung disease.

## Introduction

The majority of cases of community acquired pneumonia (CAP) are caused by bacteria as demonstrated by the dramatic decrease in mortality rates following the introduction of antibiotics^1^. Aetiological studies of CAP are however unable to define a pathogen in half of patients and this proportion falls to less than a quarter in clinical practice^2,3^. Our understanding of the bacterial aetiology of CAP is limited by the practical difficulties in obtaining timely specimens and the selectivity of sputum culture. Aetiological studies have been heavily influenced by positive blood cultures from bacteraemic patients who represent an important, but small, proportion of all those with this syndrome. These diagnostic limitations mean most patients receive the same broad spectrum, empirical antibiotic treatment despite wide variations in prior comorbidity, prior medications and age. It is likely that the bacteria which cause CAP vary in relation to patient characteristics and an understanding of these associations would guide stratified prescribing.

Culture independent molecular techniques enable the detection of low abundance and less easily cultured bacterial species and some techniques can identify species with greater accuracy than culture. For example Wilkinson *et al*. used PCR of the *S*. *pneumoniae lytA* gene to show that 64% of isolates identified as *S*. *pneumoniae* using traditional methodology following sputum culture had been misidentified and once sequenced were shown to be mostly *S*. *pseudopneumoniae*^4^. Respiratory microbiota studies demonstrated that 16S rRNA gene sequencing can characterise the bacterial composition of respiratory specimens from healthy subjects and those with respiratory disease^5,6^.

Very few studies have used next generation sequencing to investigate the respiratory microbiota of patients at the time of CAP and they have lacked the clinical detail required to advance antibiotic policy^7,8^. Sputum remains the most practical airway specimen in non-ventilated CAP and molecular testing has the potential to surmount some of the diagnostic limitations of culture. We determined the microbiota in sputum samples obtained from a large series of adults with carefully phenotyped CAP. Our aim was to inform trials of stratified, empiric antibiotic therapy by determining how clinical characteristics predict sputum microbiota in this population.

## Results

### Patient characteristics

169 patients were recruited to the study of whom 86 provided a sputum sample. 9 samples did not contain mucopurulence and were discarded leaving seventy seven sputum samples to be sequenced. The characteristics of those 77 patients are detailed in Table 1. 16.7% of patients had serological evidence of a recent influenza infection at the time they presented with CAP. 5/74 (6.8%) of patients grew *Streptococcus pneumoniae* in their blood culture. Chronic lung disease is prevalent among our population and half of the cohort self-reported a pre-existing respiratory comorbidity. COPD was three times as common as asthma and there was one case of interstitial lung disease (ILD). Radiological or physiological investigations to distinguish COPD, asthma and ILD were not performed and since these sub-groups were small, we analysed chronic lung disease as a single comorbidity.

**Table 1:** Patient characteristics.

Characteristic		N = 77 (except for*)
Age (years), median (IQR)		68 (49–76)
Male, N (%)		42 (55.3)
*Smoking status, N (%)	Active	38 (50.7)
	Quit	27 (36)
	Never	10 (13.3)
Charlson Comorbidity Index, N (%)	0	23 (30.3)
	1	32 (42.1)
	2	8 (10.5)
	3	10 (13.2)
	4	2 (2.6)
	5	1 (1.3)
	6	0
Prior pulmonary disease	Any, N (%)	46 (59.7)
	COPD, N (%)	35 (45.5)
	Asthma, N (%)	10 (13.0)
	Other, N (%)	1 (1.3)
CURB65, N (%)	0	22 (28.6)
	1	13 (16.9)
	2	23 (29.9)
	3	18 (23.4)
	4	1 (1.3)
	5	0
Prior statin use, N (%)		23(29.9)
*CRP (mg/ml), median (IQR)		147 (78.8–230.2)
*PCT (ng/ml), median (IQR)		0.77 (0.18–4.15)
*BMI (Kg/m^2^), median (IQR)		25.9 (22.3–30.2)
*Influenza infection, N (%)		9 (16.7)
*Pneumococcal bacteraemia, N (%)		5 (6.8)
Prior antibiotics, N (%)		7 (9.1)
In hospital mortality, N (%)		2 (2.6)
Length of stay (days), median (IQR)		5 (4.0–8.0)
30 day re-admission, N (%)		8 (10.4)

The Charlson comorbidity index is a tool for predicting the risk of mortality during admission that is attributable to pre-existing comorbid conditions.

CURB-65 is a validated tool for predicting risk of 30 day mortality from CAP. The components are C = confusion (present scores 1, absent scores 0), U = urea (>7 mmol/L scores 1, ≤7 mmol/L scores 0) R = respiratory rate (≥30 scores 1, <30 scores 0) B = Blood-pressure (systolic <90 mmHg or diastolic ≤60 mmHg score 1) and age (≥65 years scores 1, <65 years scores 0).

CRP is serum C-reactive protein level, <5 mg/mL is normal.

PCT is serum procalcitonin level, serum levels >5 ng/mL are strongly associated with bacterial infection and this is the threshold for antibacterial treatment being ‘strongly recommend’^43^.

BMI is body mass index.

Prior antibiotics records the number of patients who had been taking antibiotics in the community for greater than 24 hours prior to admission.

*Data was incomplete for variables marked with an asterisk: Smoking status N = 74, CRP N = 76, PCT N = 69, BMI N = 67, Influenza N = 54 and Pneumococcal bacteraemia N = 74.

### Bacteria detected by 16S rRNA gene sequencing

Considering all 77 sequenced sputum samples together, 4947 different bacterial operational taxonomic units (OTUs) were detected. The complete data-set is available through GenBank accession number PRJEB20580. As anticipated, many OTUs were rare, being found in only one sample and had very low relative abundance and we therefore performed the analysis on a sub-set of the data with these rare OTUs removed. The final data-set contained 60 OTUs of interest.

**Figure 1:**
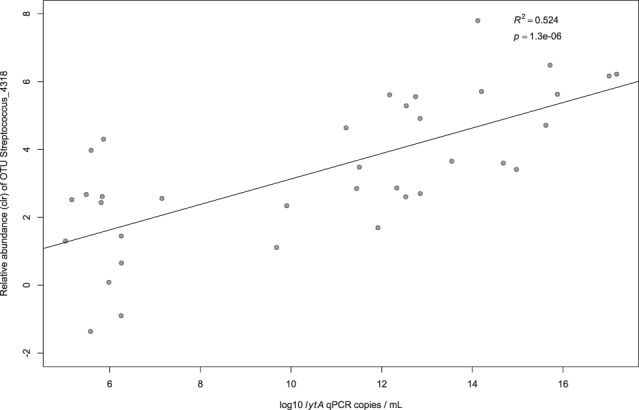
Correlation between the relative abundance of Streptococcal OTU_4318 and the concentration of *S*.*pneumoniae* in sputum. Here we compare, on the y axis, the relative abundance of the Streptococcal OTU_4318 in each sputum sample (expressed as the centred log ratio (clr)) with, on the x axis, the concentration of *Streptococcus pneumoniae (S*. *pneumoniae*) in genome copies/mL as measured by qPCR of the *lytA* gene.

Comparing all sputum samples, an airway commensal bacterium of the genus *Veillonella* spp. (Veillonella_1328) was on average the most relatively abundant^9^. Relative abundance is expressed here as the centred log ratio (clr). The clr is a statistically robust method for transforming compositional data in order to compare proportions, and the method for calculating the clr is explained in detail in the methods section. A negative clr value means that the OTU is less abundant than the mean abundance of all OTUs in that sample; a value of 0 indicates its abundance is the same as the mean abundance of OTUs in the sample and a positive value means that it is more abundant than the mean abundance of the OTUs in that sample. The second most relatively abundant OTU was a *Streptococcus* sp. (Streptococcus_4318). We compared the relative abundance of this and all other OTUs of the genus *Streptococci* spp. with the concentration of *S*. *pneumoniae* in sputum expressed as genome copies per millilitre (mL) and measured by qPCR of the *lytA* gene. Figure 1 shows that the relative abundance of the OTU Streptococcus_4318 was strongly associated with the concentration of *S*. *pneumoniae* in each sputum sample; no such association was found with any other Streptococcal OTU.

**Figure 2:**
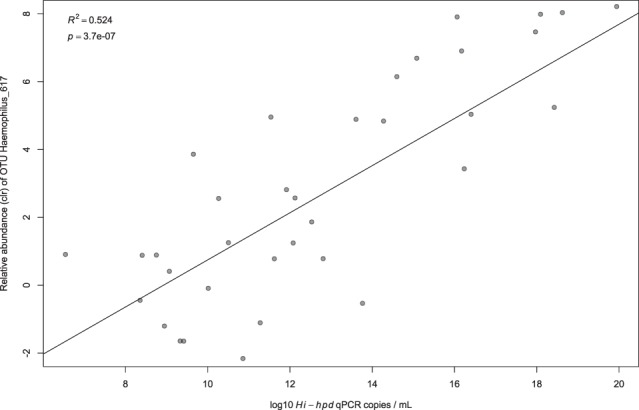
Correlation between the relative abundance of Haemophilus OTU_617 and the concentration of *H*.*influenzae* in sputum. Here we compare, on the y axis, the relative abundance of the Haemophilus OTU_617 in each sputum sample (expressed as the centred log ratio (clr)) with, on the x axis, the concentration of *Haemophilus influenzae (H*. *influenzae*) genome copies/mL as measured by qPCR of the *Hi-hpd*.

Similarly, we found a strong association between the most relatively abundant *Haemophilus spp*. OTU (Haemophilus_617) and the concentration of *H*. *influenzae* in sputum as measured by qPCR of the *Hi-hpd* gene (Fig. 2). We found no association between the relative abundance of any other *Haemophilus spp*. OTUs and the concentration of *H*. *influenzae* in sputum. Since *H*.*influenzae* can be difficult to distinguish from *H*. *haemolyticus* we compared the relative abundance of the dominant OTU Haemophilus_617 with the concentration of *H*. *haemolyticus in sputum as measured by* qPCR of the *Hh-hpd* gene and no association was found. These strong associations between species-specific qPCR results and the relative abundance of particular OTUs are highly suggestive of, but do not prove, species level identification of these OTUs.

### Associations between bacterial composition of sputum and patient characteristics

**Figure 3:**
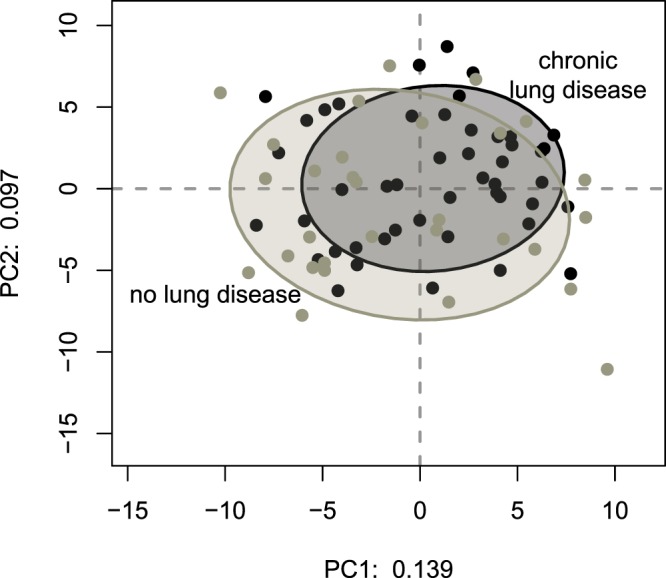
The bacterial composition of sputum samples from patients with CAP differ in the presence or absence of chronic lung disease. This plot enables us to visually compare the extent to which the bacterial composition of sputum samples differ. The axes are the first two principal components of variation in the bacterial composition of these sputum samples. The axes have no units. Black dots are sputum samples from patients with chronic lung disease and grey dots are sputum samples from patients without chronic lung disease. The ellipses encompass 75% of samples from each group and show those from patients with chronic lung disease were more closely related to one another, with a tight cluster in the top right quadrant, than those from patients with no prior lung disease. This suggests there was a bacterium or bacteria whose relative abundance was frequently similar in patients with chronic lung disease.

**Figure 4:**
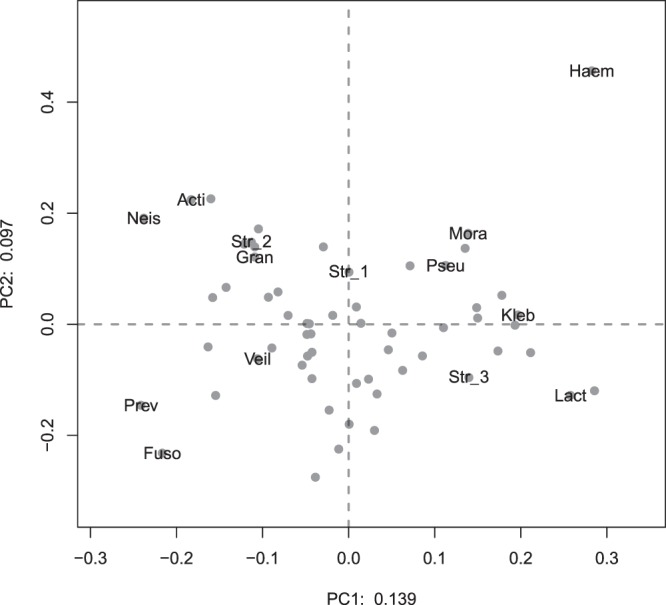
The relative abundance of bacterial OTUs in sputum. The axes are the same as in Fig. 3, but here we compare the extent to which the relative abundance of individual OTUs varies in this data-set. Each dot is a unique bacterial OTU. Certain OTUs, from genera known to cause pneumonia, have been labelled by the genus to which they belong (see key below). The position of each OTU on the plot gives an indication of how its relative abundance in samples varies. Those which are close to the origin, where the dashed-lines intersect, have a similar relative abundance in all samples (zero variance). Those which are far away from the origin are much more abundant in some samples than others. OTUs which are distant from one another, for example Haemophilus_617 and Prevotella_956, have a reciprocal pattern of relative abundance such that when one is high the other is low. OTUs which are close e.g. Pseudomonas_3976, Moraxella_2510 and Klebsiella_1954 all tend to have a higher relative abundance in the same sample. Key: Fuso = Fusobacterium_1252. Prev = Prevotella_956. Veil = Veillonella_1328. Gran = Granulicatella_740. Neis = Neisseria_4683. Acti = Actinomyces_3641. Pseu = Pseudomonas_3976. Mora = Moraxella_2510. Kleb = Klebsiella_1954. Lact = Lactobacillus_2480. Haem = Haemophilus_617. Str_1 = Streptococcus_4318. Str_2 = Streptococcus_1024. Str_3 = Streptococcus_360.

We used multivariate analysis of variance (MANOVA) to determine if clinical characteristics known on admission were associated with different patterns of bacteria in the sputum (Table 2). Chronic lung disease had the only significant association (*P* = 0.03) on the bacterial composition of sputum (shown graphically Fig. 3). To determine which bacteria made the greatest contribution to the differences in bacterial composition we conducted a principal components analysis of the OTUs found in sputum. Figure 4 shows that Haemophilus_617 was responsible for driving much of the variation in sputum composition among this cohort, with the OTU being dominant in some samples and only a minor component of others.

**Table 2:** Associations between clinical variables and the bacterial composition of sputum in CAP.

Variable	*P*
Chronic lung disease	**0.03**
Pro-calcitonin	0.14
Neutrophil count	0.15
Age	0.18
Prior statin use	0.18
Body mass index	0.19
Smoking	0.36
CURB-65 score	0.40
Charlson comorbidity index	0.42
C-reactive protein	0.59
Index of multiple deprivation	0.71
Influenza	0.78
Gender	0.92
Prior antibiotics	Not tested

The table shows the results of a Multivariate analysis of variance (MANOVA). *P* values are the result of the Wilks Lambda test comparing the variation in the relative abundance of all 60 bacterial OTUs (dependent variables) between clinical groups (independent variables e.g. gender). Too few patients had received pre-hospital antibiotics to perform a valid statistical test.

**Figure 5:**
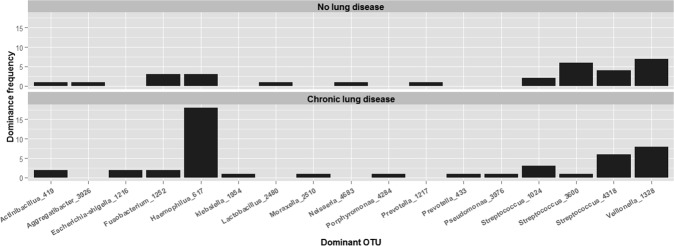
During CAP, a Haemophilus sp. is the most frequently dominant OTU in sputum from patients with chronic lung disease. In this histogram the height of each column represents the number of times a particular bacterial OTU was the most relatively abundant (dominant) OTU sequenced from sputum. Patients are divided into those with and without chronic lung disease.

Having so far shown above that a) the composition of bacteria in sputum varied in association with clinical characteristics and b) the OTU Haemophilus_617 was responsible for the greatest proportion of this variation in bacterial composition we next sought to determine which bacteria were responsible for the specific compositional changes seen in sputum from patients with chronic lung disease. We used Fischer’s exact test to determine whether the relative abundance of OTUs were associated with the presence or absence of chronic lung disease. Patients with chronic lung disease were more likely to have a high relative abundance of Haemophilus_617 than those without chronic lung disease (OR = 5, CI 1.2–29.6, *P* = 0.015). No other bacterial OTU was statistically significantly associated with presence or absence of chronic lung disease. Figure 5 illustrates this finding and demonstrates that sputum from patients with chronic lung disease was more likely to be dominated by Haemophuilus_617 than sputum from patients without lung disease.

## Discussion

We have conducted an innovative and detailed study of adult sputum microbiota in patients with acute community acquired pneumonia. The factor which had the greatest effect on the bacterial composition of sputum from adults with CAP was the presence of chronic lung disease. Modern molecular techniques gave compelling evidence that sputum from patients with chronic lung disease was more likely to be dominated by *Haemophilus influenzae* than by other bacteria. A *Streptococcus* OTU was the second most relatively abundant bacteria in these sputum samples and associated with the level of *Streptococcus pneumoniae*. However, whilst this *Streptococcus* was frequently the dominant pathogen, its relative abundance could not be predicted by patient characteristics.

The key strength of this study is that our patients were rigorously characterised using an extensive range of clinical and laboratory parameters. We used compositional sequence analysis without rarefaction, comparing proportions rather than absolute read counts, thus avoiding the associated false-positive conclusions^10,11^. It was not our intention to evaluate the use of such sequencing techniques in clinical practice but rather to apply this platform to better understand how the community structure of sputum varies in CAP in association with particular patient characteristics. This is the first microbiota study to demonstrate how a particular patient characteristic – the presence of lung disease – has an appreciable association with the bacterial community structure of sputum in patients with CAP. The relevance of this finding is that is can be tested by a randomised controlled trial since the taxonomic group driving the community structure, Haemophilus spp., suggests a particular therapeutic option.

Our cohort had similar characteristics to other prospectively recruited UK CAP cohorts^12^ however we excluded patients with malignancy and advanced dementia and it would be worth investigating individuals with these conditions. Moreover, these results only apply to patients with CAP who produce sputum and future studies should attempt to describe the bacterial microbiota in non-expectorating adult patients by bronchoalveolar lavage or percutaneous aspiration of consolidated lung^13^. Many sputum samples were dominated by a single highly abundant species suggesting pathogenic overgrowth and implying causality, but the link between relative abundance and causality is not straightforward and some invasive bacteria may be able to cause disease at lower relative abundance than others. Establishing causation with certainty may not be possible but testing the hypothesis that some bacteria are more likely to be the cause than others in certain contexts could be tested by large randomised controlled trials of different antibiotic regimes. The association of the dominant *Haemophilus* OTU with qPCR levels of *H*. *influenzae* is not proof of species identification but in the context of the known association between Nontypeable *Haemophilus influenzae* (NTHi) and lung disease this correlation is highly suggestive^14^.

Some have questioned the utility of a molecular analysis of sputum for the study of CAP on the basis of potential ‘contamination’ by upper airway bacteria. However, owing to the close correlation between bacterial populations at the two sites, several authors have recently shown that molecular analysis of sputum can provide valuable diagnostic information in CAP^15–17^. Further, we agree with the conceptual model expounded by Dickson *et al*. who highlight that, given the anatomical continuity of the respiratory tract, there are no rigid upper and lower tract communities as bacteria constantly transition throughout^18^. During pneumonia, conditions in the lung change in such a way as to enable certain bacteria to flourish. In this context, sputum remains a valid sample for analysis and features in all contemporary CAP clinical guidelines.

In current clinical practice, the culture of *H*. *influenzae* or *S*. *pneumoniae* from purulent sputum provided by a patient presenting with CAP, would be regarded as diagnostic and the patient would receive targeted antibiotics – despite the fact that both bacteria are known to colonise the upper airway of asymptomatic individuals. Moreover, the bacterial culture results reported by a clinical laboratory do not include all bacteria grown but mention only those felt to be relevant based on relative speed and quantity of growth on a range of media. The clinical culture report is therefore based on interpreting the range of cultured bacteria on the basis of relative abundance and in the light of the clinical context. This is the rationale we have applied to the interpretation of causality with our sequencing results.

We identified patients who were infected with influenza virus at the time of their CAP, but did not find an association with the relative abundance of any particular bacterial OTU. Although our primary aim was to investigate the bacteria in CAP we chose to detect influenza virus and include it as a variable since the association between influenza infection and risk of development of secondary bacterial CAP is well known. In recent studies molecular testing has demonstrated a broad range of viruses can be detected at the time of CAP, particularly in children, although their role in the disease is unclear^3^. Future studies designed to investigate the role of viruses in CAP could replicate our analysis to determine if there are associations between particular viruses and bacterial OTUs in CAP.

In comparison with the existing literature, the spectrum of bacteria identified with high relative abundance in this study was consistent with prior descriptions of pneumonia in high income countries suggesting that, the high rate of negative pathogen identifications in previous CAP studies is unlikely to be explained by a previously uncultured bacteria. Two smaller studies have previously studied sputum microbiota in CAP. Chen *et al*. analysed sputum from 45 Chinese CAP patients and compared them to saliva samples from healthy subjects and sputum from a separate cohort with hospital acquired pneumonia^7^. Their proof of concept study described differences between disease and health but had limited clinical applicability. Mizrahi *et al*. analysed sputum from 21 Israeli patients in critical care, however limited clinical information was provided and the diagnostic criteria for pneumonia were not defined^8^.

Regarding rates of pneumococcal identification, Said *et al*. estimated that for every case of pneumococcal bacteraemic CAP there are another 3 non-bacteraemic cases and that *S*. *pneumoniae* is responsible for 25% of all CAP cases^19^. This estimate fits our data where, of the 77 patients with sequenced sputum, five grew *S*. *pneumoniae* in the blood implying an additional fifteen non-bacteraemic cases giving an estimate of 20/77 = 25% of cases. Two important bacterial causes of CAP were not identified in our samples: *Legionella pneumophila* and *Chlamydophylla pneumoniae*. We used mechanical DNA extraction in order to detect intracellular bacteria and two members of the intracellular genus *Mycoplasma* were detected, at low relative abundance, so we are confident that if other intracellular bacteria had been present at a clinically meaningful levels of relative abundance they would have been detected.

Our data suggest that a *Haemophilus* sp. may be a more common cause of CAP in adults with chronic lung disease than has hitherto been appreciated. This is a view supported by several recent influential reviews^14,20^. If this finding were validated it would have implications for clinical efficacy and antimicrobial stewardship because current empirical CAP antibiotic regimens for non-severe CAP focus on beta-lactam/macrolide combinations and this provides sub-optimal cover for Gram-negative respiratory infections. If validated our data suggest doxycycline or a respiratory fluoroquinolone may be a better first line choice for those with non-severe CAP and chronic lung disease. This is supported by recent observational data from Spain where Nontypable *Haemophilus influenzae (NTHi)* resistance to beta-lactams was 23.2%^21^ and German data showing 11% more treatment failures in non-severe *H*. *influenzae* pneumonia treated with beta-lactam monotherapy compared to a tetracycline or fluoroquinolone^22^.

In conclusion, in hospitalised adults with non-severe CAP, the presence of chronic lung disease predicts the frequent dominance of a *Haemophilus* sp. These results alone should not be used to change practice but should inform a randomised trial comparing a standard empirical antibiotic regime with another expanded to cover Nontypable *Haemophilus influenzae* in patients who develop CAP on a background of chronic lung disease.

## Methods

### Patients

Between February 2011 and March 2013 we recruited consecutive patients aged ≥16 years with CAP (British Thoracic Society definition^23^) from two hospitals in Liverpool, UK. We excluded patients admitted within the last 14 days, with Cystic Fibrosis (CF) or non-CF bronchiectasis, who were immunocompromised, mechanically ventilated, required renal replacement therapy, or had either thoracic malignancy or advanced cancer of any type, those who were treated palliatively and those with severe cognitive impairment.

### Ethics and regulatory compliance statement

This work was approved by the UK NHS Research Ethics Committee (NHS REC Number 10/WNo03/40) and was listed on the NIHR Clinical Research Network portfolio. All subjects provided informed consent and, in accordance with the UK mental capacity act, a consultee provided assent on behalf of those who lacked capacity as a consequence of CAP related delirium - with consent being retrospectively obtained upon recovery of capacity. All aspects of the research were conducted in accordance with local and national policies as articulated by the UK Research Governance Framework for Health and Social Care 2005.

### In-hospital management and study procedures

The study team had no role in clinical management which was part of a regional pneumonia performance audit^24^. Recruitment and sampling occurred within 24 hours of the first dose of in-hospital antibiotic. Sputum was expectorated into sterile (pre-sealed and UV irradiated) pots and frozen immediately to −85 °C without further processing. Only samples with obvious mucopurulence were stored – those which appeared to be predominantly salivary were discarded. We recorded clinical variables which may predict different patterns of sputum bacteria e.g. socioeconomic status (see below), the Charlson comorbidity index^25^, body mass index (BMI), pro-calcitonin, C-reactive protein, neutrophil count, age, prior-medications, influenza infection and the CURB-65 score [C (confusion) U (Urea) R (Respiratory rate) B (blood pressure) age over or under 65 years)]^26^.

### Socioeconomic status

Previous studies have suggested socioeconomic status is a risk factor for the development of CAP. We used the Index of Multiple Deprivation (IMD) to describe socioeconomic deprivation and tested if socioeconomic status was an independent predictor of the pattern of bacteria present in sputum during CAP. https://www.gov.uk/government/uploads/system/uploads/attachment_data/file/465791/English_Indices_of_Deprivation_2015_-_Statistical_Release.pdf

### Influenza serology

Serum samples were analysed by haemagglutination inhibition for the following influenza viruses: B/Brisbane/60/2008, B/Wisconsin/1/2010, A/Perth/16/2009(H3N2), A/Victoria/361/2011 (H3N2), A/California/7/2009(H1N1) as previously described^27^ with minor modifications: using 4 haemagglutinating units (4HAU) and 0.5% guinea pig for influenza H3N2 and 0.5% turkey erythrocytes for influenza B and A(H1N1)pdm09. Serum samples were tested in duplicate at an initial dilution of 1:10 and a final dilution of 1:1280. Suitable control serum samples were included in all analyses; post-infection ferret serum samples raised against the five vaccine strains used in the analysis, served as positive controls, and human serum samples from individuals with either high antibody titres to currently circulating influenza H1, H3, and B strains or from individuals with no antibody titres to these seasonal strains were used as positive and negative controls, respectively. Patients were recorded as having CAP associated with an influenza infection if they had a 4 fold rise in their influenza titre between admission and follow-up.

### DNA extractions

Sputum samples were defrosted in batches and DNA was mechanically extracted using an MPBio FastPrep®-24 bench-top homogeniser and FastDNA™ SPIN Kits for Soil (MPBio http://www.mpbio.com/product.php?pid=116560200) in accordance with the manufacturer’s instructions.

### 16S rRNA gene pyrosequencing and 16S rRNA gene quantitative PCR

Polymerase chain reactions (PCR) of the V3-V5 regions of the 16S rRNA gene were performed using Q5^®^ High Fidelity Master Mix (New England BioLabs Inc.) in a 25 µl PCR including 1 µl of template DNA, 1 µl forward primer 357 F (5′CCGTCAATTYMTTTRAGT3′) and 1 µl reverse primer 926 R (5′CCTACGGGAGGCAGCAG3′). A positive and a negative PCR control was included in all PCRs and extraction kit controls were included. Reaction steps for the 16S rRNA PCRs were: −95 °C for 2 minutes then 35 cycles of 95 °C for 20 seconds then 50 °C for 30 seconds then 72 °C for 5 minutes. Four replicates of each polymerase chain reaction (PCR) were pooled to improve the detection of low abundance OTUs^28^. Once pooled the final dsDNA amplicon concentrations from each sample were quantified using the Quant-iT™ kit (Invitrogen) according to the manufacturer’s protocol. Equimolar concentrations of amplicon from each sample were then collected into a single tube creating an admixture of PCR products from all sputum samples. This meta-sample was then purified using Agencourt^®^ AMPure^®^ XP bead kits before being re-quantified using Quant-iT™ (Invitrogen). The purified amplicon pool underwent pyrosequencing on a 454 Life Sciences GS FLX (Roche Diagnostics, Oakland, CA) as per the manufacturer’s protocol. 16S rRNA gene qPCRs used the 357 F and 926 R primers (as above) in 10 ml triplicate quantitative PCRs (qPCRs) incorporating KAPA SYBR FAST Universal 23 qPCR master mix (Kapa Biosystems, Woburn, MA), and analysed the data with Corbett rotor-gene 6.1 software (Qiagen, Venlo, the Netherlands) as previously published^29^.

### Analysis of sequence data

Pre-processing of the raw sequencing data was performed using the Quantitative Insights into Microbial Ecology (QIIME) pipeline for analysis^30^. Within QIIME AmpliconNoise was used to de-noise and de-multiplex the samples and Perseus removed chimeras^31^. Operational taxonomic units (OTUs) were assigned by clustering sequences at a 97% identity threshold in UCLUST program and the most abundant sequence within an OTU cluster was chosen as the representative sequence for that OTU^32^. The Silva SSU Ref ND database (version 111) was used for taxonomical assignment of each OTU by using an 80% bootstrap confidence around a representative sequence from each OTU^33^. PyNAST was used for alignment of representative sequences by accessing the Silva reference set^34^. FastTree was used for phylogenetic tree reconstruction from a representative sequence of each OTU^35^.

Subsequent analyses involving assigned OTUs were performed in R (ver3.3.2) using the compositional analysis package CoDaSeq (https://github.com/ggloor/CoDaSeq). For each sample, sequence read counts were transformed by taking the log of the ratio of the count for an OTU and the geometric mean of the counts of all OTUs in that sample; this transformation is termed the centred log ratio (clr)^36^. The clr values use log 2; a clr of 5 therefore means the OTU was 32 fold more abundant than the mean abundance of all OTUs in that sample. High throughput sequencing data is ‘sparse’ meaning it contains many zeros as a consequence of rare bacteria. Methodologies for handling sparse data are an area of ongoing research but we chose to replace zeros using a Bayesian approach using the “CZM” method of the ‘cmultRepl’ function within the R package zCompositions^37^. Since our aim was to study how variation in bacterial composition might relate to clinical covariates such as comorbidity, we filtered the data to remove non-informative OTUs whose clr value varied between samples less than the median variance of all OTUs; the clr transformation permits this approach by preserving correlation and variance properties when the data is sub-set^10^. This work was performed concurrently with the work of Salter *et al*. who demonstrated that commercial DNA extraction kits may be contaminated by bacterial DNA leading to spurious conclusions unless accounted for; as a consequence extraction kit controls were included in our sequencing runs^38^. We observed contaminating OTUs associated with the DNA extraction kit used on a particular date and this was most obvious in low biomass samples from another study. Given that similar extraction kits were used for the sputum samples in this study a sensitivity test was performed by repeating the planned analysis with and without those potentially contaminating OTUs; the inclusion or exclusion of these OTUs had no effect on the associations between OTUs and clinical characteristics nor on the relative abundance of OTUs. Principal components analysis (PCA) was used to explore which samples were compositionally similar and which clinical characteristics explained any clustering of samples. Multivariate analysis of variance (MANOVA) along with the Wilks lambda test was implemented to determine statistical significance of observed differences.

### Pathogen specific quantitative PCR (qPCR)

For certain genera 16S rRNA gene amplicon sequencing, due to high levels of homology, is unable to discriminate to the level of individual species. As this was the case for both *Streptococcus* spp. and *Haemophilus* spp., genera that contain important respiratory pathogens, quantitative PCRs (qPCRs) were performed^39^. Detection and quantification of *S*. *pneumoniae* DNA was performed by *lytA* qPCR using the following primer and probe sequences: forward primer, 5′-ACGCAATCTAGCAGATGAAGCA-3′; reverse primer, 5′-TCGTGCGTTTTAATTCCAGCT-3′; probe, 5′-(6-FAM)-TGCCGAAAACGCTTGATACAGGGAG-(BHQ-1®)-3′. Each PCR reaction contained 12.5 ul of Taqman gene expression mastermix (Applied Biosystems, California, USA), 900 nM of each primer, 500 nM of the probe and 2.5 ul of extracted DNA and the standard curve consisted of six serial dilutions (10^6^–10^1^) of lytA plasmid (Fast-Track Diagnostics, Slierna, Malta). Thermal cycling was performed in a StepONE system (Applied Biosystems) under the following conditions: 10 min at 95 °C followed by 40 cycles of 15 sec at 95 °C and 1 min at 60 °C.

*Haemophilus influenzae* DNA was detected and quantified by qPCR of the *H*. *influenzae hpd* gene (*Hi-hpd*) using the *hpd*#3 PCR of Wang *et al*.^40^ with the following modifications: both primers were used at a concentration of 300 nM and the probe was labelled with FAM dye and BHQ1 quencher; the PCR was duplexed with an inhibition detecting positive control based on Murphy *et al*.^41^. This comprised the published gfpF and gfpR primers, published gfp probe labelled with CY5 dye and BHQ2 quencher, plus purified genomic DNA from an *E*. *coli* strain engineered to carry the *gfp* gene from *Aequorea victoria* (*E*. *coli* BLR-GFP). Each final PCR reaction contained Taqman Universal PCR master Mix (Applied Biosystems, UK), 300 nM each of hpdF822 and hpdR952 primers, 100 nM hpdPb896i (FAM/BHQ1) probe, 150 nM each of gfpF and gfpR primers, 200 nM gfp (CY5/BHQ2) probe, 1 pg *E*. *coli* BLR-GFP DNA, plus 2 µl of sample DNA extract in a final volume of 25 µl. PCR was performed on a 7500 Fast Real-Time PCR System (Applied Biosystems, UK) using the manufacturer’s recommended cycling conditions. Ct values were converted to DNA concentrations using a standard curve of purified *H*. *influenzae* genomic DNA (Vircell, Spain) spanning 2.5 to 10000 genome copies/µl.

*Haemophilus haemolyticus* was detected and quantified by qPCR specific for the *H*. *haemolyticus hpd* gene (*Hh-hpd*) using a variation of the method of Latham *et al.*^42^. Each 20 µl reaction contained PowerUp SYBR Green Master Mix (Applied Biosystems, UK), 300 nM of Hh-*hpd* F and Hh*-hpd* R primers. PCR was performed on a 7500 Fast Real-Time PCR System (Applied Biosystems, UK) using the manufacturer’s recommended cycling conditions. Ct values were converted to DNA concentrations using a standard curve of purified *H*. *haemolyticus* (NCTC10659) genomic DNA spanning 10 to 10000 genome copies/µl. The *H*. *haemolyticus* genomic DNA was prepared using the QIAsymphony SP automated instrument (QIAGEN), quantified using the Qubit dsDNA BR Assay Kit (Life Technologies, Paisley, UK) and converted to genome copies/µl using a value of 2.0fg/genome.
